# Tinnitus outcomes after cochlear implantation in single-sided deafness: an exploratory prospective multicenter study

**DOI:** 10.3389/fneur.2026.1970581

**Published:** 2026-09-28

**Authors:** Jae-Jin Song, Yeonjoo Choi, Yehree Kim, Jun Ho Lee, Hong Ju Park, Jong Woo Chung

**Affiliations:** 1 Department of Otorhinolaryngology-Head and Neck Surgery, Seoul National University Hospital, Seoul National University College of Medicine, Seoul, Republic of Korea; 2 Department of Otorhinolaryngology-Head and Neck Surgery, Asan Medical Center, University of Ulsan College of Medicine, Seoul, Republic of Korea

**Keywords:** cochlear implantation, pitch matching, single-sided deafness, tinnitus, tinnitus handicap inventory

## Abstract

Tinnitus is thought to be suppressed through partial reversal of auditory deafferentation via cochlear implantation (CI). By stimulating the deafferented auditory pathway, multiple studies have revealed that tinnitus is significantly suppressed in 66–92% of patients undergoing CI. This study aimed to evaluate the efficacy of CI as a treatment for tinnitus and to investigate post-CI audiologic outcomes in patients with single-sided deafness (SSD). From November 2018 to July 2020, 10 patients who were diagnosed with SSD and suffering from tinnitus were prospectively recruited at three tertiary referral hospitals. Participants in this study agreed to undergo CI as a treatment for tinnitus. Postoperative aided pure tone audiometry (PTA) and word recognition score (WRS) were measured. Questionnaires related to tinnitus, such as the tinnitus handicap inventory (THI) and visual analogue scale (VAS) scores including loudness, distress, and perception, were administered. Additionally, a tinnitogram was obtained for loudness and pitch matching. Audiological outcomes of CI were analyzed in conjunction with the results of pitch-matching from the tinnitogram. All 10 patients completed device-on evaluations, and 9 patients completed device-off evaluations. THI scores showed statistically significant improvement 3 months after CI in both the device-on and device-off conditions (preoperative: 78; device on: 38, *p* = 0.027; device off: 50, *p* = 0.049). VAS scores also demonstrated significant improvement after CI (preoperative: 8.2; device on: 3.3, *p* = 0.011; device off: 6.5, *p* = 0.012). Four patients demonstrated changes in psychoacoustic tinnitus features after CI: 2 changed to lower frequency, 1 changed to higher frequency, and 1 patient’s tinnitus changed from white noise to a specific identifiable frequency. There was no significant tendency for threshold (T-level and C-level) changes in pitch-matching frequency. In line with previous literature, this exploratory multicenter study demonstrated improvement in tinnitus loudness and distress after CI in patients with single-sided deafness and tinnitus, with a tendency for greater reduction in tinnitus intensity observed when the device was activated. Additionally, approximately half of the patients showed changes in the psychoacoustic characteristics of tinnitus after CI. Given the small sample size and the absence of a non-implanted control group, these findings should be regarded as exploratory and hypothesis-generating, and warrant confirmation in adequately powered controlled trials.

## Introduction

Single-sided deafness (SSD) refers to unilateral severe-to-profound sensorineural hearing loss, in which the contralateral hearing threshold is normal or near-normal (≤30 dB) (1). Even when contralateral hearing is normal, subjects with SSD suffer from impaired sound localization and speech perception in noisy backgrounds (2, 3). Individuals with acquired unilateral hearing loss experience diminished quality of life due to the loss of sound localization, difficulty in speech perception in noisy environments, and challenges in understanding conversations. Psychological issues and limitations in social engagement, such as anxiety about potential hearing loss in the unaffected ear, moments of embarrassment linked to hearing difficulties, and decreased self-confidence in one’s participation abilities, can result in the experience of social isolation (4, 5). Tinnitus is one of the most common symptoms in people with hearing loss and is frequently debilitating in patients with acquired SSD (6). Therefore, people with an acquired SSD need auditory rehabilitation even though their contralateral hearing is normal.

Cochlear implantation (CI) is the most effective method of auditory rehabilitation in severe-to-profound hearing loss patients. For individuals with unilateral hearing loss who cannot benefit from hearing aids or other devices, CI is a very promising solution (7, 8). Contralateral routing of signal (CROS) hearing aids and bone conduction devices (BCD) can also be options for auditory rehabilitation for SSD; however, these two devices do not supply the necessary auditory input from both ears in SSD patients, which is essential for true binaural hearing (9). In contrast, CI not only aids in hearing restoration but also provides assistance with directional hearing (10).

In 2008, Van de Heyning et al. (11) first performed CI on patients with SSD and tinnitus, and reported significant tinnitus reduction. The researchers discovered a notable and reliable decrease in the intensity of tinnitus, leading them to propose CI as a novel approach for addressing tinnitus caused by SSD (11). Moreover, other studies have also demonstrated successful outcomes of tinnitus suppression by CI in SSD patients (12–14). A long-term outcome study of up to 10 years of CI in SSD patients showed that CI is beneficial not only for tinnitus reduction but also for hearing improvement (15). More recent evidence has further consolidated CI as a treatment option for SSD. In a two-year prospective cohort of 70 adults with SSD, tinnitus severity decreased markedly by 6 months after CI and remained reduced thereafter, although tinnitus persisted in a substantial proportion of patients (16); in a secondary analysis of the same cohort, tinnitus burden was the most consistent negative correlate of hearing-related quality of life, and early postoperative tinnitus burden was associated with quality-of-life outcomes at 2 years (17). A Nordic cohort study (18) and a prospective multicenter post-market study (19) have likewise evaluated speech recognition in noise, sound localization, quality of life, tinnitus, and safety after CI in adults with SSD, and recent reviews discuss the extension of CI indications to this population (20). In most of these cohorts, however, CI was indicated for hearing rehabilitation and tinnitus was assessed as one of several outcome domains; prospective data in which tinnitus is the primary indication for surgery and the psychoacoustic characteristics of tinnitus are tracked in relation to device programming remain scarce. While there have been several studies on the successful reduction of tinnitus through CI, there has been no fundamental understanding of the mechanism by which CI can suppress tinnitus. Various factors, including masking, direct electrical stimulation of nerves, habituation, and plastic reorganization in the brain, need to be taken into account when exploring possible explanations (21). It is conceivable that restoring sound-evoked activity to the deprived auditory pathway may reverse the maladaptive changes underlying tinnitus generation. Therefore, in individuals experiencing tinnitus alongside hearing impairment, CI may also offer relief of tinnitus symptoms (11, 14).

Assuming that tinnitus is suppressed through electrical stimulation of the cochlea via CI, one might raise questions about whether specific electrical channels have an impact on tinnitus suppression. Di Nardo et al. (22) aimed to identify specific channels demonstrating efficacy in tinnitus suppression among CI patients. The results indicated that a suppressive channel could be identified in 38% of the patients. In other words, the study suggested the potential for tinnitus suppression through targeted electrical stimulation in CI patients. Arts et al. (23) suggested that tinnitus suppression could be achieved through intracochlear electrical stimulation in patients with SSD. In a subsequent study, they showed that tinnitus could be suppressed not only by the recovery of environmental sound but also by intracochlear stimulation (24). Therefore, different modes of electrical stimulation may induce different levels of tinnitus suppression.

Therefore, we aimed to investigate the efficacy of CI in patients with SSD with regard to tinnitus improvement, to examine how the perceived tinnitus frequency changes after surgery, and to explore mapping-threshold changes at the pitch-matched frequency after CI. Given the small cohort and the absence of a non-implanted comparison group, the present study was designed as an exploratory, hypothesis-generating investigation rather than a confirmatory trial.

## Materials and methods

### Study design and patients

This prospective study was conducted from November 2018 to July 2020 on patients who had been diagnosed with SSD and were suffering from tinnitus in three tertiary hospitals.

The inclusion criteria for this study were: (1) Adult patients, 19 years or older, with acquired severe unilateral hearing loss [pure tone audiometry (PTA) threshold >70 dB HL] for at least 6 months, (2) Patients who developed severe tinnitus [visual analogue scale (VAS) loudness > 7] after the onset of acquired severe unilateral hearing loss, (3) Patients who voluntarily decided to participate in this study with an understanding of CI, (4) Patients for whom participation in a 1-year follow-up observation after study enrollment was feasible, (5) Individuals who provided voluntary informed consent by signing the informed consent form, (6) Patients deemed suitable for CI based on computed tomography (CT) scan results of the temporal bone. The threshold of VAS loudness >7 was chosen deliberately. Because CI is an invasive and irreversible procedure that, in this study, was offered primarily for tinnitus relief rather than for hearing rehabilitation alone, the study protocol restricted enrollment to patients with severe tinnitus, in whom the anticipated benefit was judged to outweigh the surgical risk. This approach is in line with earlier studies of CI for tinnitus in SSD, which likewise enrolled only patients with incapacitating tinnitus (11, 13). A severe baseline also reduced the risk of floor effects when quantifying post-CI change. Accordingly, SSD patients with mild-to-moderate tinnitus (VAS loudness ≤ 7) were not enrolled, and the present findings therefore pertain to SSD patients with severe tinnitus and should not be generalized to the broader SSD population with milder tinnitus (see Limitations).

All included patients underwent preoperative hearing and tinnitus tests including inventories. Upon examination, the side of deafness and tinnitus, duration of deafness and tinnitus, and preoperative tympanic membrane status were evaluated. All patients underwent CI surgery under general anesthesia without any complications. The implanted device was the Neuro system (Oticon Medical), comprising the Neuro Zti implant and the Neuro One sound processor.

Patients were followed up at 1, 3, 6, and 12 months after surgery. The study protocol was approved by the Institutional Review Board (IRB) of the institutes in three different tertiary hospitals (IRB numbers, 2017–1,389; B-1804-465-006; 1810–079-979).

### Evaluation of hearing thresholds

Hearing thresholds were determined by PTA, and the mean hearing levels were expressed as the average of the hearing thresholds at 0.5, 1, 2, and 4 kHz (four-frequency average). The term “scale out” is defined as having no response to sounds ranging from 0.25 kHz at 100 dB, 0.5 kHz at 115 dB, 1 kHz at 120 dB, 2 kHz at 115 dB, 4 kHz at 115 dB, to 8 kHz at 105 dB. To understand the changes in hearing ability after CI, functional gain (FG), which is aided PTA after CI, was obtained, and word recognition score (WRS) was also obtained from subjects, pre- and post-operatively. FG and WRS were measured preoperatively, and at 1, 3, 6, and 12 months after surgery.

### Evaluation of tinnitus

Questionnaires such as Tinnitus Handicap Inventory (THI), and Visual Analog Scale (VAS) scores including loudness, distress, and perception were measured preoperatively, and at 1, 3, 6, and 12 months after surgery.

THI consists of 25 questions including 11 functional subscale items, 9 emotional subscale items, and 5 catastrophic subscale items. Patients answered yes (4 points), sometimes (2 points), and no (0 points) for each question, and the total scores were measured (25). On the VAS, patients rated loudness, distress, and perception from 0 to 10 according to their subjective perception. Both questionnaires were conducted when the device was on and off.

In addition, a tinnitogram was obtained for loudness and pitch matching, measured through the contralateral ear. In pitch matching, the patient was instructed to determine the pitch of their tinnitus, which was then matched to a tone generated by the audiometer. In loudness matching, the tinnitus loudness was compared to a tone of the same frequency, generated by the audiometer (26). This tinnitogram was planned to be conducted in 4 different conditions: (1) Condition 1: all-electrodes off, (2) Condition 2: tinnitus pitch-matched single electrode-on, otherwise off, (3) Condition 3: basal electrodes (high frequency range) on, otherwise off, (4) Condition 4: full electrodes-on, standard stimulation pattern with environmental sound perception (27).

### Mapping

Mapping of CI was conducted with a software program named ‘Genie Medical CI.’ Twenty electrodes comprised the CI device; from 1 (high frequency, around 7 kHz) to 20 (low frequency, around 0.25 kHz) (Table 1). The default frequency allocation of each electrode is provided in the footnote to Table 1. T-level, which is the initial current level perceived as sound when gradually increasing the current amplitude, and C-level, which is the current amplitude that is gradually increased to a level where the sound is perceived as loud and comfortable without causing discomfort, were measured through the program. Patients underwent mapping at 1, 3, 6, and 12 months after surgery. Mapping outcomes of CI were analyzed with the results of pitch matching in tinnitogram.

**Table 1:** Subjects mapping results.

		1 month	3 months	6 months	12 months
Subject No 1. Pitch matching: 3 kHz to 0.25 kHz					
Electrode 6 (3 kHz; 2,688–3,313 Hz)	T level	12	11	13	16
	C level	34	41	44	53
Electrode 20 (0.25 kHz; 188–313 Hz)	T level	10	10	10	11
	C level	14	33	38	37
Average of threshold in electrode except 6 and 20	T level	10.22	14	15.94	17.33
	C level	31.5	50	55.56	57.67
Subject No 2. Pitch matching: 8 kHz to 6 kHz					
Electrode 1 (8 kHz; 6,688–7,938 Hz)	T level	11	12	12	13
	C level	54	59	50	45
Electrode 2 (6 kHz; 5,563–6,688 Hz)	T level	10	10	10	11
	C level	48	57	49	46
Average of threshold in electrode except 1 and 2	T level	16.56	17.72	18	16.94
	C level	53.89	59.5	58	55.67

T and C levels are expressed in device current units. Default frequency allocation of the 20-electrode array (Neuro Zti, Oticon Medical): E1, 6,688–7,938 Hz; E2, 5,563–6,688 Hz; E3, 4,688–5,563 Hz; E4, 3,938–4,688 Hz; E5, 3,313–3,938 Hz; E6, 2,688–3,313 Hz; E7, 2,313–2,688 Hz; E8, 1938–2,313 Hz; E9, 1,688–1938 Hz; E10, 1,438–1,688 Hz; E11, 1,313–1,438 Hz; E12, 1,188–1,313 Hz; E13, 1,063–1,188 Hz; E14, 938–1,063 Hz; E15, 813–938 Hz; E16, 688–813 Hz; E17, 563–688 Hz; E18, 438–563 Hz; E19, 313–438 Hz; E20, 188–313 Hz.

### Statistical analysis

Continuous variables are expressed as mean and standard deviation or median and range, while categorical variables are expressed as number and percentage. Variables were compared using the Wilcoxon signed-rank test. A *p* value of less than 0.05 was considered significant. Statistical analyses were performed using IBM SPSS Statistics, version 24.0 (IBM Corp., Armonk, NY, USA).

## Results

### Patient demographics

In total, 10 patients were enrolled in this study. The demographics of each patient are summarized in Table 2. The median age of the patients was 59.5 years (range, 51–78), and the median duration of hearing loss was 4.8 years (range 0.6–12.6). There were 4 cases of hearing loss in the right ear and 6 cases in the left ear. Tinnitus occurred bilaterally in 2 patients and only in the ear with hearing loss in the remaining 8 patients. The median duration of tinnitus was also 4.8 years (0.6–12.6), which was the same as the duration of hearing loss. There was no inflammation in the middle ear and mastoid cavity in any of the patients.

**Table 2:** Demographics of the patients.

No	Age (year)	Sex	Single side deafness side	Single side deafness duration (year)	Tinnitus side	Tinnitus duration (year)	Preoperative PTA (dB HL)	Preoperative WRS (%)	Postoperative final aided PTA (dB HL)	Postoperative final aided WRS (%)
1	56	M	Right	2	Both	2	73.75	40	40	52
2	64	M	Left	10	Both	10	80	32	43.75	52
3	60	F	Right	4.6	Right	4.6	86.25	4	38.75	No data***
4	51	M	Left	7.5	Left	7.5	72.5	40	35	20
5	58	M	Right	9	Right	9	67.5	4	No data	No data
6	52	F	Left	5	Left	5	Scale out*	CNT**	48.75	64
7	78	M	Left	12.6	Left	12.6	91.25	4	55	12
8	59	F	Left	0.6	Left	0.6	Scale out	0	46.25	76
9	75	F	Right	0.7	Right	0.7	Scale out	0	41.25	56
10	71	F	Left	1.5	Left	1.5	77.5	28	32.5	No data
Median (range)	59.5			4.8		4.8	77.5	4	41.25	52

^*^Scale out refers to unresponsiveness at the maximum output of the audiometer for air conduction (0.25 kHz, 100 dB HL; 0.5 kHz, 115 dB HL; 1 kHz, 120 dB HL; 2 kHz, 115 dB HL; 4 kHz, 115 dB HL; 8 kHz, 105 dB HL). In patients #6, #8, and #9, no response was obtained at any of the four frequencies (0.5, 1, 2, and 4 kHz) used for the four-frequency average.

^**^CNT, cannot test.

^***^No data indicates that the corresponding assessment was not performed because the patient missed the corresponding follow-up visit.

### Audiologic changes after CI

All patients underwent aided PTA and WRS after CI to evaluate hearing thresholds with the device. Table 2 demonstrates the preoperative PTA and aided PTA after CI. The median value of aided PTA improved to 41.25 dB HL (range, 32.5–55.0) after surgery compared to the preoperative score of 77.5 dB HL (range 67.5–91.25). Aided WRS with CI was also evaluated, and the score reached 52% (range, 12–76) compared to the preoperative score of 4% (range, 0–40).

### Tinnitus changes after CI

Among the 10 patients, all underwent THI evaluation with the device ‘on’, and 1 patient did not undergo THI evaluation with the device ‘off’. With the device on, an improvement in THI scores was observed in 9 patients (Figure 1a). One patient exhibited worsening results when the device was on. Similar outcomes were noted when the device was turned off (Figure 1b). On average, THI scores steadily improved after surgery, and the differences were statistically significant from 3 months after surgery in both the device-on and device-off conditions (*p* = 0.027 and *p* = 0.049, respectively). Both device on and off conditions were significant; however, the ‘on’ status demonstrated greater effectiveness in reducing tinnitus (Figures 1a,b).

**Figure 1:**
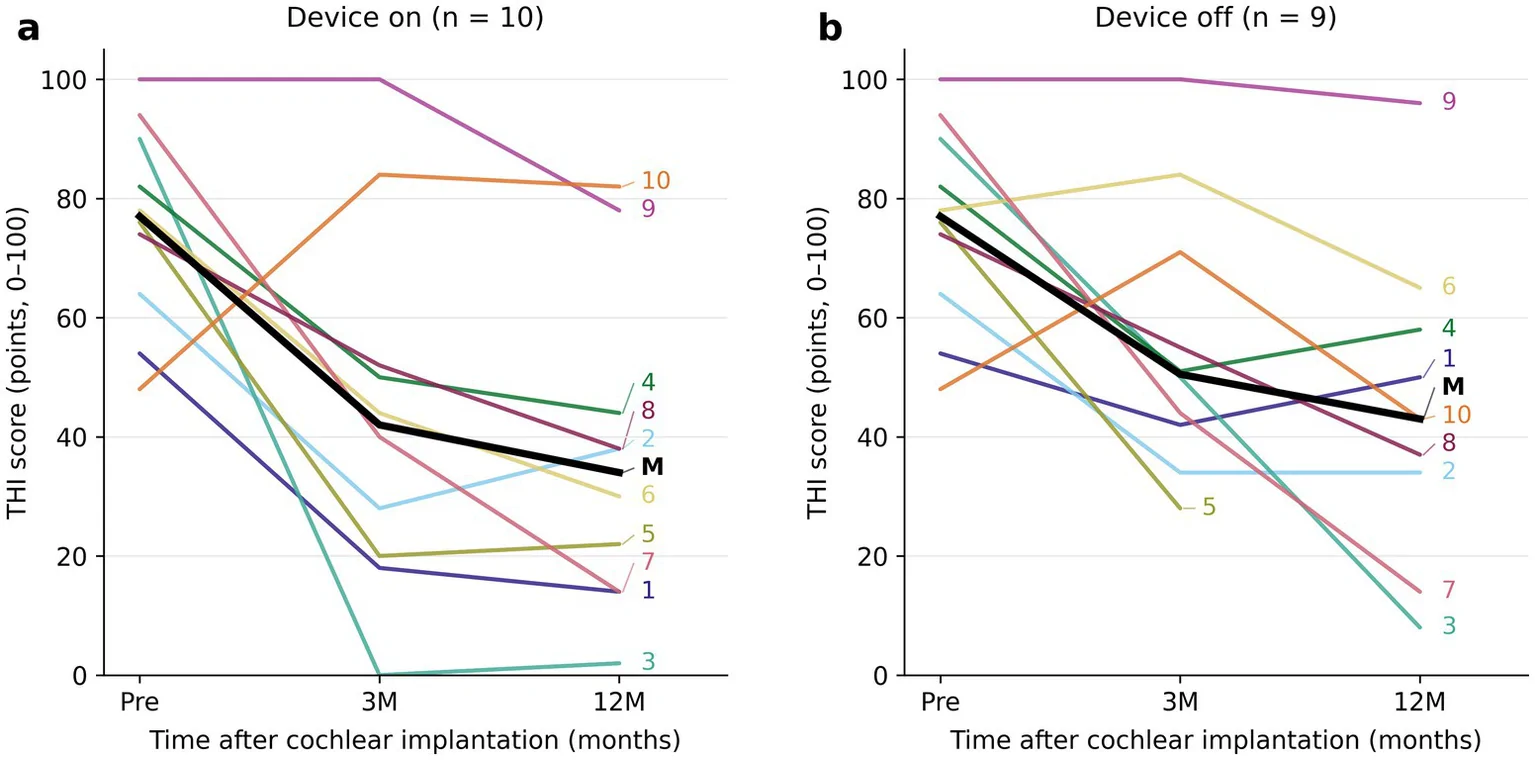
Individual and median Tinnitus Handicap Inventory (THI) scores before (pre) and 3 and 12 months (3M, 12M) after cochlear implantation (**a**: device on, *n* = 10; **b**: device off, *n* = 9). The number at the right end of each line indicates the individual patient; M denotes the median. Note that patient 5 completed the device-off evaluation only up to 3 months.

Regarding VAS scores, loudness, distress, and perception were evaluated as subdivisions. All 10 patients underwent VAS evaluation with the device ‘on’, and 1 patient did not undergo VAS evaluation with the device ‘off’. When considering the total VAS score, all 10 individuals showed improvement in VAS scores compared to pre-surgery when the device was turned on. Improved VAS scores were also observed when the device was turned off in 9 individuals (1 individual: no data). Total VAS scores before and after surgery with on and off situations are demonstrated in Figures 2a,b. The figures demonstrated that scores after CI decreased immediately after surgery in both on and off situations (sum; *p* = 0.011, *p* = 0.012, respectively), especially more in the device on status. It was observed that subdivision into loudness, distress, and perception similarly resulted in improved scores.

**Figure 2:**
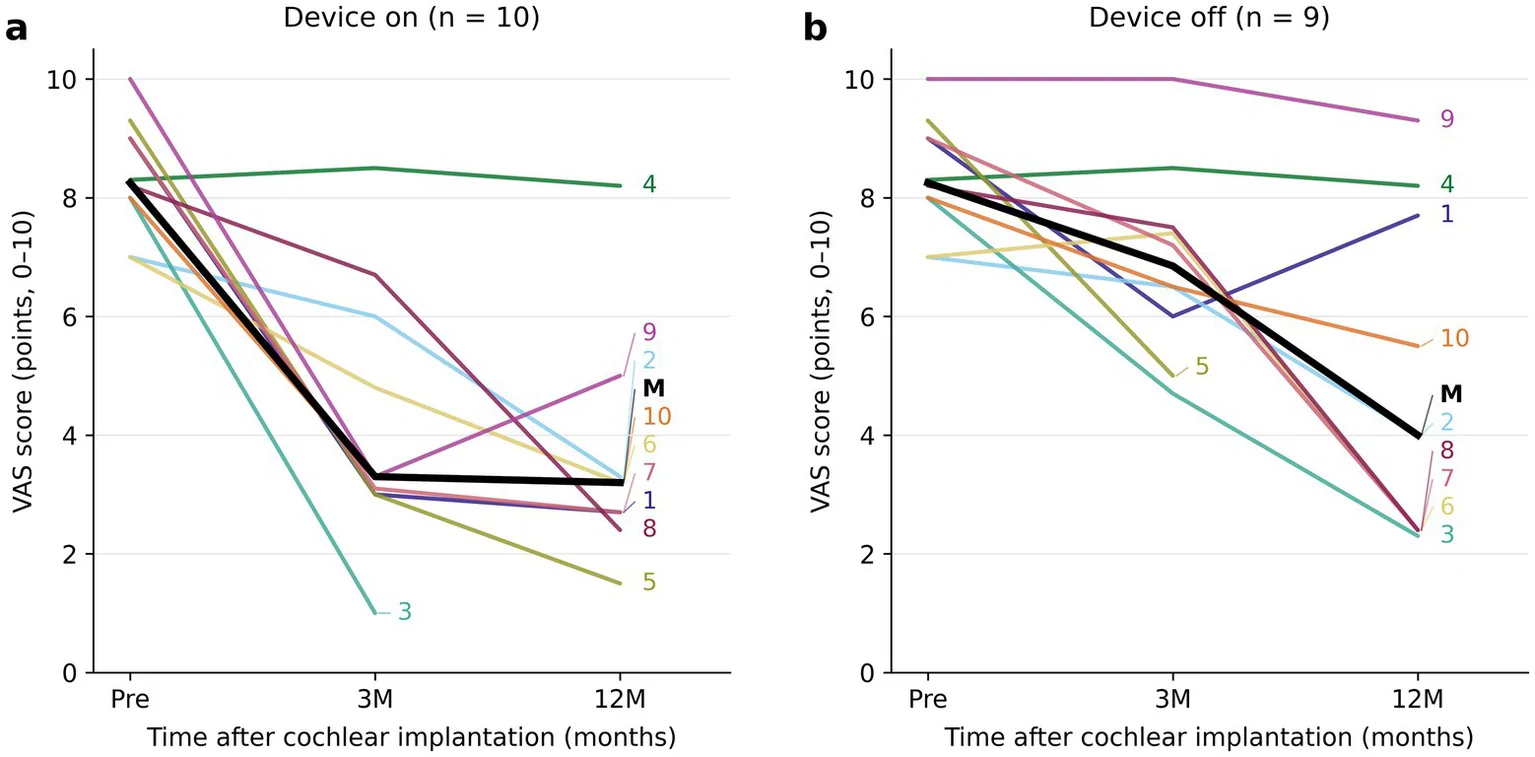
Individual and median visual analogue scale (VAS) scores before (pre) and 3 and 12 months (3M, 12M) after cochlear implantation (**a**: device on, *n* = 10; **b**: device off, *n* = 9). The number at the right end of each line indicates the individual patient; M denotes the median.

The tinnitogram consisted of 2 subdivisions: loudness and pitch. Loudness matching in the tinnitogram could be completed by 4 of the 10 patients, as the remaining patients were unable to reliably match the loudness of their tinnitus to the presented tones. Figure 3 demonstrates the changes in loudness measured in the tinnitogram, showing a decrease from 6.0 dB SL to 2.5 dB SL after surgery, although this was not statistically significant (*p* = 0.180). In pitch matching, 8 patients reported pure tone-type tinnitus, while 2 reported white noise-like tinnitus resembling the sound of wind. Among these 2 patients with white noise tinnitus, one reported a change in the psychoacoustic characteristics of tinnitus to pure tone-type after surgery. Among the 8 patients with pre-CI pure tone-type tinnitus, 3 individuals exhibited changes in the perceived tinnitus frequencies after surgery. Following CI, 2 patients experienced shifts towards lower frequencies, while 1 experienced a shift towards higher frequencies. The tinnitogram revealed variations in tinnitus frequencies under four different conditions, and only two patients were able to complete all the specified stimulation-mode conditions; these condition-specific observations are therefore purely descriptive and exploratory. One patient exhibited consistent frequencies irrespective of the conditions, while the other patient demonstrated different frequency changes based on the conditions, both at 6 months and 1 year post-surgery. At 6 months after CI, this patient showed frequency changes in conditions 2, 3, and 4 (2 kHz) compared to condition 1 (6 kHz). One year post-surgery, the patient displayed frequency changes in condition 2 (2 kHz) compared to conditions 1 and 3 as well as condition 4 (6 kHz).

**Figure 3:**
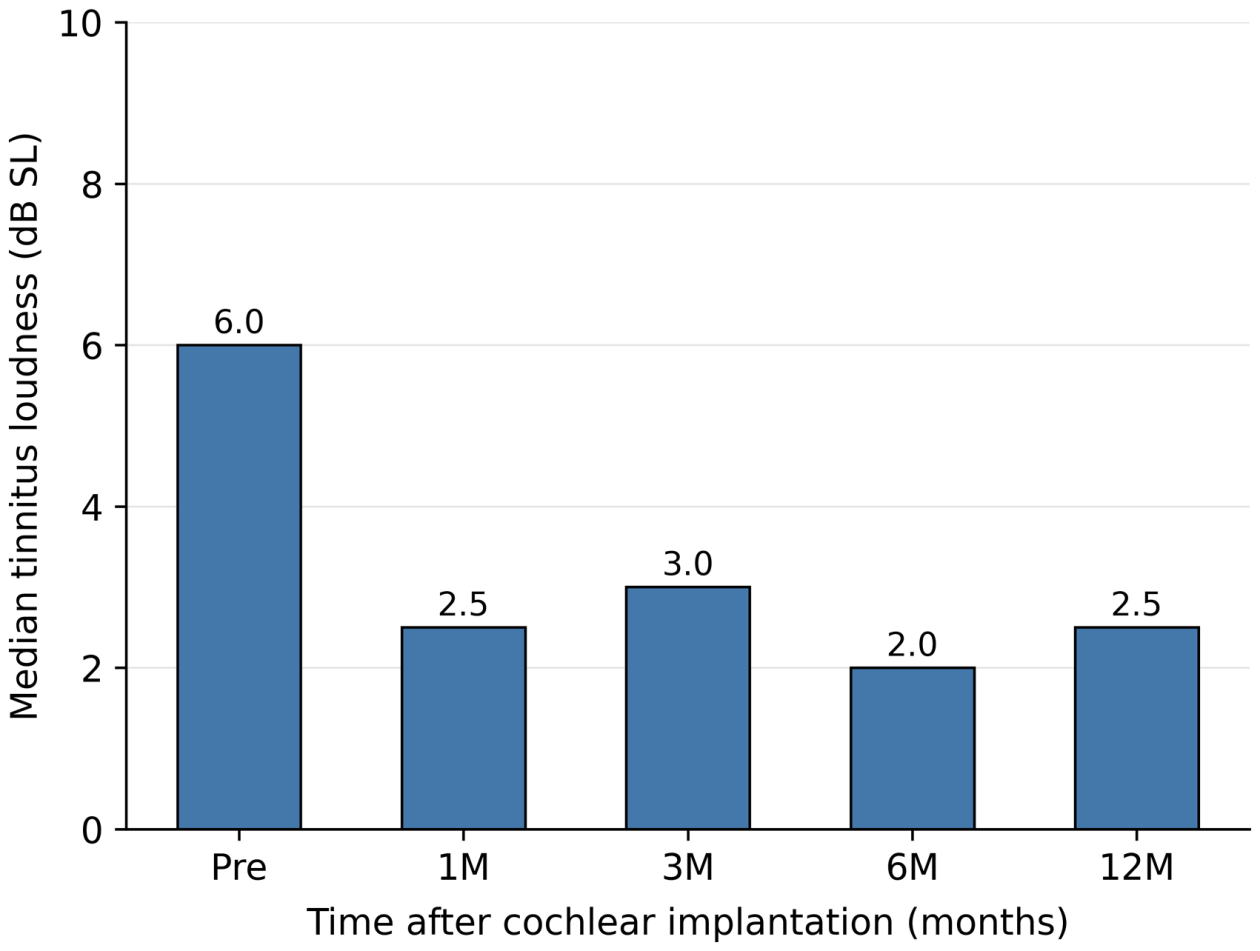
Median loudness of tinnitus (dB SL) measured by loudness matching in the tinnitogram before (pre) and 1, 3, 6, and 12 months after cochlear implantation.

### Relationship between Tinnitogram and mapping thresholds

Patients underwent mapping at 1, 3, 6, and 12 months after surgery. Only the 2 patients who completed both the pitch-matching tinnitogram and mapping were analyzed. One patient showed that their tinnitus-matched frequency had changed from 3 kHz to 0.25 kHz (Table 1). When T-level and C-level were examined for each frequency, there were no strong tendencies, but the C level of the electrode matched with 0.25 kHz had increased. When averaging the C-level and T-level of electrodes excluding electrodes 6 and 20, it was observed that the values were somewhat higher compared to electrodes exhibiting frequency changes. Another patient showed that their tinnitus-matched frequency had changed from 8 kHz to 6 kHz. T-level and C-level were examined (Table 1); however, there seemed to be no specific tendency of changing thresholds. When averaging the C-level and T-level of electrodes excluding electrodes 1 and 2, it was also observed that the values were somewhat higher compared to electrodes exhibiting frequency changes. In both patients, lower T-levels and C-levels were observed at frequencies where frequency changes occurred compared to frequencies where no changes occurred.

## Discussion

In the current study, we showed considerable improvement in hearing ability and tinnitus reduction in patients with SSD after CI. Moreover, frequency from pitch matching tinnitogram demonstrated frequency changes after CI, whereas a definite relationship between frequency change and mapping threshold change could not be established.

As tinnitus occurs due to excessive neural activity resulting from auditory deprivation, it is theoretically believed that relieving auditory deprivation through partial recovery of the peripheral auditory pathway via CI could lead to suppression of tinnitus. By stimulating the auditory pathways that have experienced peripheral deprivation using CI, studies have revealed significant suppression of tinnitus in 66–92% of patients with bilateral severe to profound hearing loss who have received CI (27). The mechanism behind this phenomenon involves the plasticity of the central auditory system including the cerebral cortex, and the provision of auditory stimulation, which can help redirect attention away from tinnitus (28).

Our results suggest a beneficial effect of CI on tinnitus, as evidenced by multiple tinnitus inventories, although the uncontrolled design of the present study precludes definitive causal inference. However, it is worth noting that one patient exhibited inconsistent responses after CI. While the THI records suggested suboptimal values when the device was turned on, improvements in tinnitus were reported in the VAS score when the device was activated. This discrepancy arose from the fact that the VAS score measured the severity of tinnitus, whereas the THI assesses the self-perceived handicap imposed by tinnitus on daily life. In other words, patients may perceive a decrease in tinnitus intensity after CI without a corresponding reduction in tinnitus-related handicap. The THI consists of a total of 25 items, comprising 11 items in the functional subscale, 9 items in the emotional subscale, and 5 items in the catastrophic subscale (28, 29). Although these subscales were not explicitly distinguished in this study, it could be speculated that the patient’s persistent complaints of dizziness both pre- and post-surgery, as well as severe tinnitus when not using the device post-surgery, may suggest that lower scores were recorded in the emotional subscale.

Many studies have also demonstrated that even in cases of unilateral hearing loss patients with severe tinnitus, CI can suppress tinnitus, establishing it to some extent as a standard treatment option when other therapies do not yield satisfactory results for SSD and debilitating tinnitus (6, 12–15, 30). One study demonstrated that tinnitus completely improved in 60% of patients using the device, while 40% of patients showed improvement only in specific situations (12). In another study, it was demonstrated that the use of the device led to a reduction in tinnitus in 100% of cases post-CI surgery compared to pre-surgery. However, when the device was deactivated, 41% of cases showed no change in the severity of tinnitus (11). Recent meta-analytical studies have reported findings indicating a subjective reduction in tinnitus through CI, supporting the efficacy of this intervention (31, 32). Our study similarly reported more effective suppression of tinnitus, especially when the device was activated, aligning with previous research. For patients experiencing worsening symptoms, further investigation is warranted to understand the underlying causes.

Mertens et al. (15) demonstrated the long-term effects of CI in patients suffering from tinnitus with SSD. They used tinnitus-related questionnaires and interviews to evaluate the effects of CI on patients and revealed significant improvement in tinnitus reduction up to 3 months after the first fitting, especially when the CI device was on, and this remained stable thereafter. Our study also demonstrated that patients subjectively reported a reduction in tinnitus within 1–3 months, with a notable decrease especially when the device was activated. A study by Fan et al. (33) demonstrated that following implantation, there was a notable reduction in the tinnitus perception, loudness, and distress compared to the period prior to implantation from 6 months after implantation, with psychological benefits. A study by Punte et al. (13) also presented findings on the effects of CIs concerning tinnitus in SSD patients. From the results of our study, CI appeared to result in improvement of tinnitus shortly after surgery, and this effect seemed to endure for an extended period. Further long-term follow-up and observation will be necessary for a more comprehensive understanding in the future. In this respect, the recent two-year prospective cohort of Schrader et al. (16) is informative: in 70 adults with SSD, tinnitus-related distress fell markedly within the first 6 months after CI and then plateaued, and approximately 80% of patients still reported some degree of tinnitus at 2 years. The temporal profile of our THI and VAS data, with most of the improvement occurring by 3 months and relative stability thereafter, is consistent with this early-improvement-then-plateau pattern, and the incomplete suppression we observed, particularly with the device off, mirrors the persistence of residual tinnitus in that larger cohort. The same group further showed that tinnitus burden was the most consistent negative correlate of hearing-related quality of life after CI, and that tinnitus burden at 6 months was associated with quality of life at 2 years (17), underscoring the clinical relevance of tinnitus-specific outcome assessment in SSD implantees. Contemporary cohorts from other regions, including a Nordic cohort study (18) and a prospective multicenter post-market study (19), have similarly included tinnitus among the outcome domains alongside speech-in-noise perception, sound localization, quality of life, and safety, and recent reviews discuss extending CI indications to adults with SSD (20). Against this background, the specific contribution of the present exploratory study lies in its focus on patients implanted primarily for tinnitus and in the longitudinal characterization of psychoacoustic tinnitus features in relation to electrode mapping, rather than in the magnitude of tinnitus reduction per se.

Of note, improvements in THI and VAS scores were also observed when the device was switched off. It should be emphasized, however, that the device-off condition does not constitute a sham or placebo control. Patients had undergone surgery, were fully aware of their implant status, and were assessed within the same treated cohort without blinding; the device-off assessment therefore differs from the device-on assessment only in the presence or absence of concurrent electrical stimulation, not in any of the non-specific factors that a true sham condition is designed to control for. The improvements seen in the device-off condition could be interpreted as a potential carry-over effect of CI, such as residual inhibition or stimulation-induced plastic reorganization of the central auditory system that outlasts the stimulation itself. However, these results may equally be driven by expectation bias or placebo responses associated with surgery and device use, which are likely to be accentuated in a self-selected cohort that had elected to undergo CI specifically for tinnitus relief, as well as by regression to the mean or the natural course of tinnitus. Because the present study did not include a non-implanted control group, the carry-over hypothesis remains speculative, and the device-off findings should not be taken as evidence of a durable, stimulation-independent treatment effect. The larger reductions in tinnitus scores with the device on than off, observed for both THI and VAS, are at least compatible with a contribution of electrical stimulation itself; however, because patients were aware of the device status during each assessment, this on–off difference is likewise susceptible to expectation effects and requires confirmation in controlled, ideally blinded, designs.

In the current study, mapping results of CI were analyzed with the results of pitch matching in the tinnitogram. We clearly observed changes in the frequency of tinnitus after implantation, but the mechanism is unclear. Targeted stimulation through the electrode could be more effective in patients with a tinnitus pitch in a specific suppressive channel (22). Although not consistently observed, the possibility of the existence of a specific suppressive channel was noted, suggesting that intracochlear electrical stimulation through CI could be effective in alleviating tinnitus in SSD patients (23). Therefore, this highlighted the need for further research and understanding of how to stimulate specific electrodes post-CI surgery. Based on the findings of the current study, although there was a change in frequency after CI, it was challenging to elucidate a distinct pattern of change. The indication that tinnitus frequency was altering suggested the possibility that stimulation in the cochlear region might influence tinnitus variations. Additional studies utilizing a larger pool of patient data are required to delve deeper into this aspect. Currently, it remains unclear how the areas of brain activation responsible for tinnitus perception change through CI and how these altered activations might lead to changes in the frequency of tinnitus. Currently, there is growing interest in the brain’s abnormal activity and connectivity changes associated with tinnitus, and numerous studies are being conducted in this area. According to a recent study by Schlee et al. (34), increased activity in the temporal cortices associated with tinnitus is interconnected within a larger network of widespread cortical connections. A study by Salvari et al. (35) also showed a network specific to the frequency of tinnitus, involving left fronto-temporal, fronto-parietal, and temporo-parietal junctions. Through these diverse studies, we anticipate the exploration of changes in brain activity caused by tinnitus and subsequent research investigating how this altered brain activity might be connected to changes in tinnitus frequency following CI.

Our study has several limitations that should be considered when interpreting the results. First, the limited number of participants posed a challenge in analyzing the effect of CI in alleviating tinnitus, especially considering the heterogeneous nature of tinnitus symptoms among patients; consequently, the scarcity of patients with specific frequency characteristics further limits subgroup interpretation. In addition, because enrollment was restricted to patients with severe tinnitus (VAS loudness > 7), the findings may not generalize to SSD patients with milder tinnitus, in whom both the baseline burden and the potential for measurable improvement are smaller. Moreover, recent work indicates that CI candidates evaluated under expanded indications differ at baseline from traditional bilateral candidates (36), so that findings from conventional CI populations cannot simply be transferred to SSD, and results from a tinnitus-selected SSD cohort such as ours cannot be transferred to the general SSD population. Second, and importantly, all participants had already elected to undergo CI as a treatment for their tinnitus at enrollment. The study therefore lacked a comparison group of SSD patients with tinnitus who chose alternative management options, such as contralateral routing of signal devices, bone conduction implants, or watchful waiting. Without such a control group, expectation effects and selection bias cannot be excluded, the unblinded within-subject device-off comparison cannot substitute for a sham or placebo control, and the central clinical question of the efficacy of CI for tinnitus in SSD cannot be definitively answered by the present dataset. Third, the tinnitogram could be completed by only a subset of patients, and the stimulation-mode conditions were assessed in only two patients; these observations should accordingly be regarded as descriptive and hypothesis-generating rather than conclusive. Finally, although this study demonstrated changes in the pitch-matched tinnitus frequency after CI, no definite relationship between frequency changes in the tinnitogram and mapping thresholds could be established. Taken together, the present findings are best viewed as exploratory trends that warrant confirmation in adequately powered controlled trials, ideally randomized comparisons of CI against alternative rehabilitation strategies in SSD patients with tinnitus.

## Conclusion

In this exploratory multicenter study, CI was associated with suppression of tinnitus and improving hearing ability in SSD patients, together with changes in the pitch-matched frequency as well as the loudness of tinnitus. In patients with a specific tinnitus frequency, we observed a shift in tinnitus pitch to a different frequency after CI. For patients reporting a narrow band noise of tinnitus, our findings indicated that the tinnitus either remained consistent in a similar pattern or transformed into tinnitus with a discernible frequency post-CI. Taken together, CI may be considered a promising treatment option for SSD patients suffering from tinnitus. However, given the small sample size and the lack of a non-implanted control group, these findings are hypothesis-generating, and confirmation in adequately powered controlled trials is warranted before CI can be firmly recommended as a tinnitus treatment per se.

## Data Availability

The raw data supporting the conclusions of this article will be made available by the authors, without undue reservation.
